# Child opportunity index is associated with pediatric firearm injury in Philadelphia, Pennsylvania

**DOI:** 10.3389/fpubh.2024.1339334

**Published:** 2024-01-24

**Authors:** Anireddy R. Reddy

**Affiliations:** ^1^Department of Anesthesiology and Critical Care Medicine, Children’s Hospital of Philadelphia, The University of Pennsylvania Perelman School of Medicine, Philadelphia, PA, United States; ^2^PolicyLab, Children’s Hospital of Philadelphia, Philadelphia, PA, United States; ^3^Leonard Davis Institute of Health Economics, University of Pennsylvania, Philadelphia, PA, United States

**Keywords:** geospatial analysis, child opportunity index, pediatric, firearm injury, social determinants of health

## Abstract

**Introduction:**

Firearm injury is the leading cause of death in children. This study uses geospatial mapping to illustrate the burden of pediatric firearm injury in Philadelphia and assesses the relationship between Child Opportunity Index (COI) and injury, hypothesizing that lower COI zip codes would have higher injury and mortality rates.

**Methods:**

Pediatric firearm injury data for children aged 0–19 years in Philadelphia, from 2015 to February 2023, was visualized by race/ethnicity, fatal versus non-fatal status, and COI for zip code. COI was then dichotomized as “High” or “Low” based on nationally normed scores and used to compare incidence and odds of mortality. Injury incidence rates by COI were calculated using weighted Poisson regression, to adjust for the total number of children in each COI category. Odds of mortality by COI, adjusted for age, sex and race/ethnicity, were calculated using multivariable logistic regression.

**Results:**

Of 2,339 total pediatric firearm injuries, 366 (16%) were fatal. Males (89%), adolescents (95%) and Black children (88%) were predominately affected. Geospatial mapping showed highest burden in North and West Philadelphia, which corresponded with areas of low COI. The incidence rate ratio (IRR) of injury in low COI zip codes was 2.5 times greater than high COI (IRR 2.5 [1.93–3.22]; *p* < 0.01). After adjusting for age, sex, and race/ethnicity, odds of mortality in low COI zip codes was nearly twice that of high COI zip codes (aOR 1.95 [0.77–4.92]), though did not demonstrate statistical significance (*p* = 0.16).

**Conclusion:**

Child opportunity index is associated with pediatric firearm injury in Philadelphia, Pennsylvania.

## Introduction

Firearm injury is the leading cause of death in children (1). There are over 7,000 children affected annually (2), a number that has increased dramatically in the last few years (1, 3). The burden of firearm injury is intimately related to social determinants of health and physical environment, resulting in inequalities in firearm-related deaths by homicide, suicide, or accidental injury (4, 5). Geospatial analysis is one strategy to further understand the unequal distribution of firearm injury and has primarily been used to characterize adult firearm injury, with emerging pediatric data (6–9). Prior studies have shown associations between firearm injury and lack of green space (10), vacant lots (11, 12), different policing practices (13), and social vulnerability (14–16). However, these studies have focused primarily on adult populations, which have a different distribution of injury intent and mechanism compared to children (17), and do not account for particular neighborhood factors (such as proximity to childcare and schools) which may promote child health. The aim of this study was to use geospatial mapping to illustrate the burden of pediatric firearm injury in Philadelphia, Pennsylvania and to determine the relationship between a pediatric-specific measure of social determinants of health—the Child Opportunity Index (COI)—and pediatric firearm injury. The hypothesis was that zip codes with low COI would have higher incidence of pediatric firearm related injury and mortality.

## Methods

Pediatric firearm injury data was publicly available from the Office of the Controller for the City of Philadelphia (18). Data for children aged 0–19 years was obtained from 2015 (earliest data available) through February 2023. Pediatric firearm injury and death were visualized by race/ethnicity, fatal vs. non-fatal status, and COI for zip code. Zip code data was missing for seven children. COI is an area-based index of social determinants of health for children (19) and was also available publicly. COI creates a score based on 29 indicators across education, health/environment, and socioeconomic domains (19). Each indicator is converted to a z-score (0–100), averaged, and then categorized into Very Low, Low, Moderate, High and Very High Opportunity based on nationally normed scores. Because of low numbers of injuries in the higher COI categories, COI was subsequently dichotomized into “High” or “Low” based on nationally normed z-score (0–49 for “Low” and 50–100 for “High”) for regression analysis. Firearm injury (fatal and non-fatal) incidence rates by COI were calculated using weighted Poisson regression, in order to account for the total number of children in each COI category. The association between COI and fatal pediatric injuries was assessed using multivariable logistic regression, adjusting for age, gender, and race/ethnicity. Geospatial analysis was completed using ArcMap (ArcGIS Desktop: Release 10. Redlands, CA: Environmental Systems Research Institute). Standard descriptive statistics, Poisson regression, and logistic regression were completed in Stata (StataCorp. Stata 17. College Station, TX).

## Results

**Figure 1:**
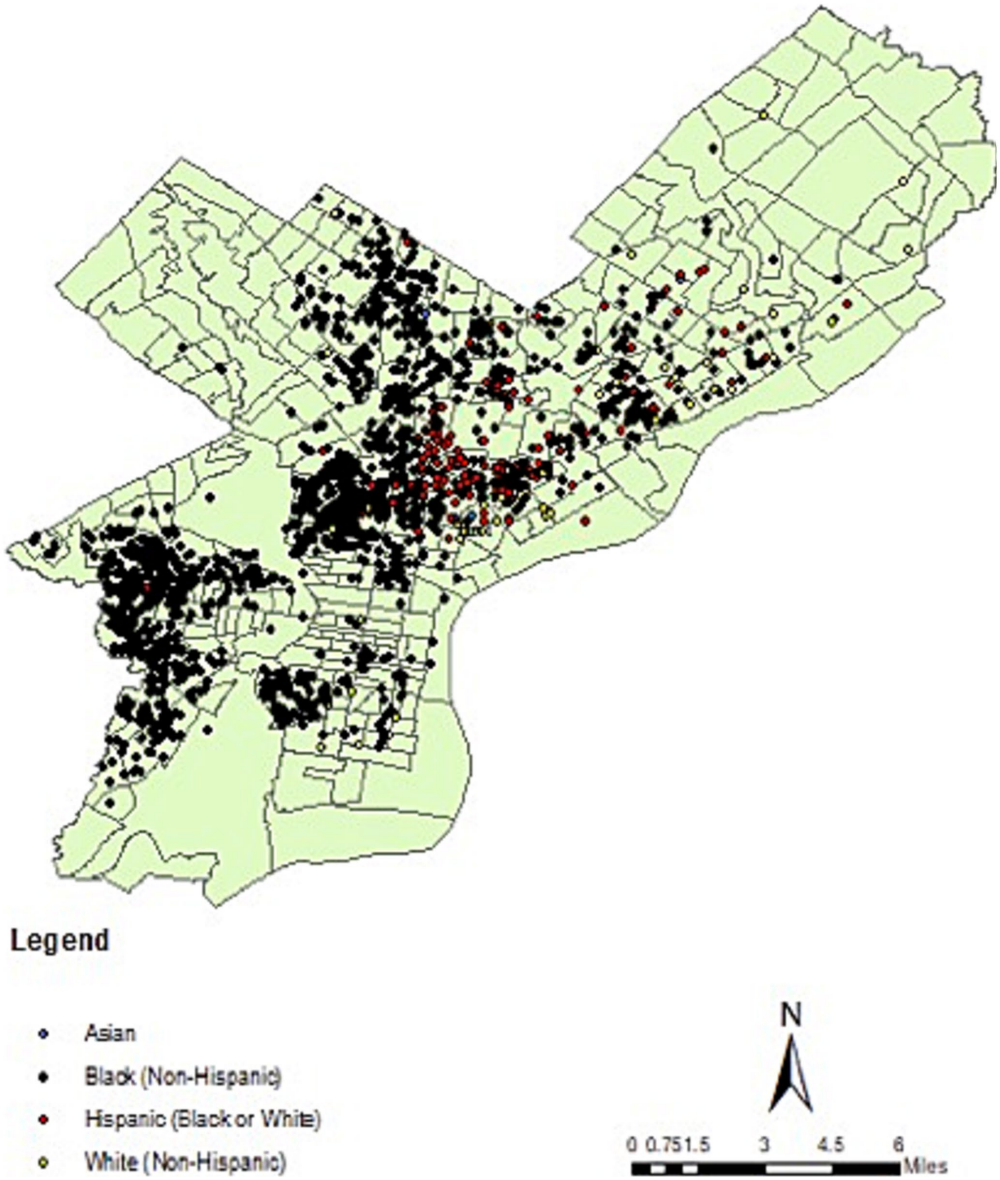
Pediatric firearm injury (fatal and non-fatal) in Philadelphia by race/ethnicity, from 2015 to 2023.

**Figure 2:**
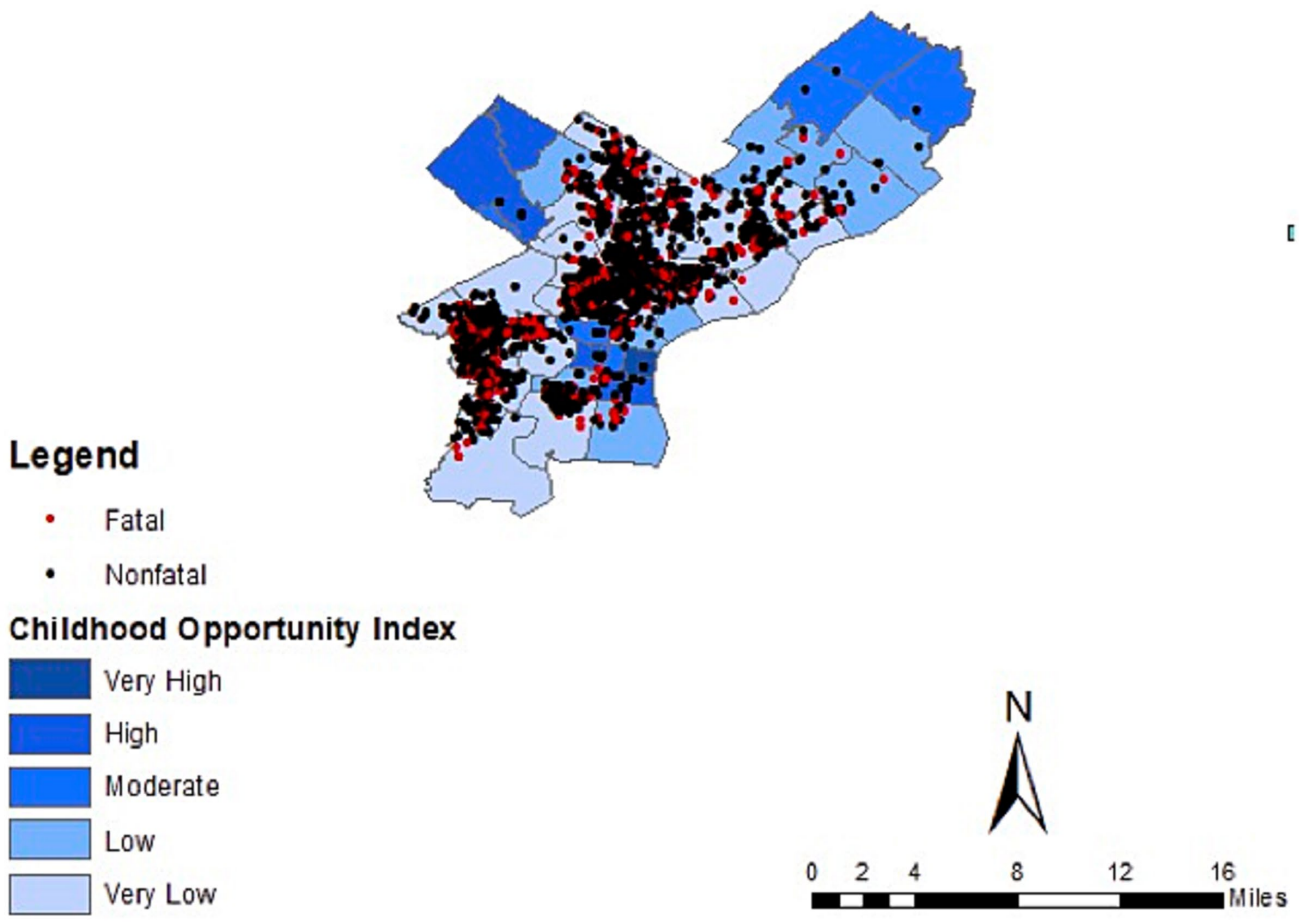
Pediatric firearm injury and death by child opportunity index in Philadelphia, from 2015 to 2023.

During study period, there were 2,339 pediatric firearm injuries, 366 (16%) of which were fatal (Table 1). Males were affected more (89%) compared to females (11%) and almost all (95%) were adolescents (13–19 years of age). Non-Hispanic Black children were predominately affected (88%) followed by Hispanic (9%), White (3%) and Asian (<1%) children. Nearly all the injuries and deaths occurred in the Low and Very Low COI quintiles (98% of all injuries, 96% of deaths). Geospatial mapping showed highest burden of both injuries and deaths in North and West Philadelphia, predominately affecting Black and Hispanic children (Figure 1), which also corresponded with areas of low COI (Figure 2). The incidence rate ratio (IRR) of injury in low COI zip codes was 2.5 times greater than high COI zip codes (IRR 2.5 [1.93–3.22]; *p* < 0.01; Table 2). After adjusting for age, sex, and race/ethnicity, odds of mortality in low COI zip codes was nearly twice that of high COI zip codes (aOR 1.95 [0.77–4.92]), though did not demonstrate statistical significance (*p* = 0.16; Table 2).

**Table 1:** Characteristics of children with firearm injury in Philadelphia 2015–2023.

	Fatal	Nonfatal	Total
	*N* = 366 (16%)	*N* = 1,973 (84%)	*N* = 2,339 (100%)
Age			
0–5 years	10 (3%)	34 (2%)	44 (2%)
6–12 years	9 (2%)	59 (3%)	68 (3%)
13–19 years	347 (95%)	1,880 (95%)	2,227 (95%)
Sex			
Male	342 (93%)	1,735 (88%)	2,077 (89%)
Female	24 (7%)	238 (12%)	262 (11%)
Race/Ethnicity			
White (Non-Hispanic)	13 (4%)	49 (2%)	62 (3%)
Black (Non-Hispanic)	313 (86%)	1,737 (88%)	2,050 (88%)
Hispanic (Black or White)	38 (10%)	181 (9%)	219 (9%)
Asian	2 (<1%)	6 (<1%)	8 (<1%)
Child Opportunity Index (Nationally Normed)			
Very High	0 (0%)	4 (<1%)	4 (<1%)
High	5 (1%)	50 (3%)	55 (2%)
Moderate	1 (<1%)	14 (1%)	15 (1%)
Low	33 (9%)	139 (7%)	172 (7%)
Very Low	325 (89%)	1,761 (89%)	2,086 (84%)
Missing	2 (<1%)	5 (<1%)	7 (<1%)

**Table 2:** Incident rate ratio and adjusted odds ratio of mortality by child opportunity index.

	Incidence rate ratio*		Adjusted odds ratio of mortality**	
Child opportunity index (dichotomized based on nationally normed z-score)	IRR [95% CI]	Value of *p*	aOR [95%CI]	Value of *p*
High	Ref	–	Ref	–
Low	2.5 [1.93–3.22]	<0.01	1.95 [0.77–4.92]	0.16

*N* = 2,332 (excludes seven patients for which COI not available). *Weighted Poisson regression to account for differences in population size in each COI category. **Adjusted for age, race/ethnicity, and sex.

## Discussion

This retrospective geospatial analysis illustrates the unequally distributed burden of pediatric firearm injury and death in Philadelphia and demonstrates an association between low COI and pediatric firearm injury. These data showed non-Hispanic Black males and adolescents as being most affected, a trend which has been observed nationally (2). The association between lower COI and pediatric firearm injury also mirrors findings from a large national retrospective study using the Pediatric Health Information System (PHIS) database (6) as well as a city-level study assessing injury in Milwaukee (7). There is notable overlap of social determinants of health, structural racism, and incidence of pediatric firearm injury. North and West Philadelphia demonstrated highest burden of firearm injury, with predominately Black and Hispanic children affected in these areas. While these areas overlap with Low or Very Low COI zip codes, race is not a full proxy for COI in this sample. After adjusting for race/ethnicity, there was nearly two-fold increased odds of firearm-related death in Low compared to High COI zip codes (Table 2), though this finding was not statistically significant. There is a wealth of literature which points to redlining, known as the historic racist lending practices of the Home Owners Loan Corporation, as the foundation for neighborhood level segregation and disparities seen today (20). At least one study found a statistically significant association between historically redlining and COI (21). With the caveat that this sample is limited, we see that COI, while co-linear with race, may contribute separately to both prevalence of firearm injury and increased odds of mortality by firearm injury.

Understanding the link between COI and pediatric firearm injury applies a child-focused lens to potentially modifiable economic, education, and health systems which may be protective against firearm injury. Further research is needed to understand what these factors may be in low COI neighborhoods and if they are amenable to intervention. While some of these factors, such as green space, walkability, and house vacancy have been associated with firearm injury and targeted for intervention (11, 12), there are other factors such as presence of early childhood education (ECE) centers, proximity to schools, toxic exposures (i.e., extreme heat exposure, industrial pollutants), food insecurity, employment rates and others which could be explored further. There are opportunities for targeted prevention efforts in areas with higher burden of injury, such as safe storage counseling (22, 23) and hospital-based violence intervention programs (24). There are additionally implications for emergency response systems and trauma care as geospatial access to care has been shown to be associated with firearm mortality in Philadelphia (25).

There are several limitations to this study. Firstly, because only zip code level data was available for the pediatric firearm injuries, it was not possible to do more granular analysis at the census-tract level. There exists heterogeneity within zip codes, and even block-to-block differences in COI, which is not accounted for in this study. Additionally, the most recent COI data is from 2020 and therefore may not be representative of the early end of the study period and cannot account for recent changes in both neighborhood exposures and trends in firearm injury during and after the COVID pandemic (3, 26, 27). This particular injury data set also does not have intent of injury (suicide, homicide, or accidental) which would be helpful to understand and develop potential interventions.

## Conclusion

Retrospective geospatial analysis of pediatric firearm injury in Philadelphia demonstrates an association between low COI and pediatric firearm injury, with children in low COI zip codes experiencing 2.5 times greater incidence of injury compared to high COI zip codes. While odds of mortality in low COI zip codes was nearly double that of high COI zip codes, it did not demonstrate statistical significance. This evidence suggests strong interplay between structural racism, social determinants of health, and burden of pediatric firearm injury and high-risk areas should be targeted for intervention.

## Data availability statement

The original contributions presented in the study are included in the article/supplementary material, further inquiries can be directed to the corresponding author.

## Author contributions

AR: Conceptualization, Formal analysis, Investigation, Methodology, Visualization, Writing – original draft, Writing – review & editing.

## References

[1] Goldstick JE Cunningham RM Carter PM. Current causes of death in children and adolescents in the United States. N Engl J Med. (2022) 386:1955–6. doi: 10.1056/NEJMc2201761, PMID: 35443104 PMC10042524

[2] Fowler KA Dahlberg LL Haileyesus T Gutierrez C Bacon S. Childhood firearm injuries in the United States. Pediatrics. (2017) 140:e20163486. doi: 10.1542/peds.2016-3486, PMID: 28630118 PMC6488039

[3] Schleimer JP McCort CD Shev AB Pear VA Tomsich E de Biasi A. Firearm purchasing and firearm violence during the coronavirus pandemic in the United States: a cross-sectional study. Inj Epidemiol. (2021) 8:43–10. doi: 10.1186/s40621-021-00339-5, PMID: 34225798 PMC8256207

[4] Kim D. Social determinants of health in relation to firearm-related homicides in the United States: a nationwide multilevel cross-sectional study. PLoS Med. (2019) 16:e1002978. doi: 10.1371/journal.pmed.1002978, PMID: 31846474 PMC6917210

[5] Buggs SA Kravitz-Wirtz ND Lund JJ. Social and structural determinants of community firearm violence and community trauma. Ann Am Acad Pol Soc Sci. (2022) 704:224–41. doi: 10.1177/00027162231173324

[6] Kwon EG Nehra D Hall M Herrera-Escobar JP Rivara FP Rice-Townsend SE. The association between childhood opportunity index and pediatric hospitalization for firearm injury or motor vehicle crash. Surgery. (2023) 174:356–62. doi: 10.1016/j.surg.2023.04.011 37211510

[7] Georgeades C Farazi M Bergner C Bowder A Cassidy L Levas MN. Characteristics and neighborhood-level opportunity of assault-injured children in Milwaukee. Inj Epidemiol. (2023) 10:43. doi: 10.1186/s40621-023-00453-6, PMID: 37605186 PMC10441698

[8] Urrechaga EM Stoler J Quinn K Cioci AC Nunez V Rodriguez Y. Geodemographic analysis of pediatric firearm injuries in Miami, FL. J Pediatr Surg. (2021) 56:159–64. doi: 10.1016/j.jpedsurg.2020.09.032, PMID: 33158506

[9] Trinidad S Vancil A Brokamp C Moody S Gardner D Parsons AA. Relationships between socioeconomic deprivation and pediatric firearm-related injury at the neighborhood level. J Trauma Acute Care Surg. (2022) 93:283–90. doi: 10.1097/TA.0000000000003679, PMID: 35546249

[10] Kondo MC South EC Branas CC Richmond TS Wiebe DJ. The association between urban tree cover and gun assault: a case-control and case-crossover study. Am J Epidemiol. (2017) 186:289–96. doi: 10.1093/aje/kwx096, PMID: 28481962 PMC5860224

[11] Moyer R Mac Donald JM Ridgeway G Branas CC. Effect of remediating blighted vacant land on shootings: a citywide cluster randomized trial. Am J Public Health. (2019) 109:140–4. doi: 10.2105/AJPH.2018.304752, PMID: 30496003 PMC6301418

[12] Sadatsafavi H Sachs NA Shepley MM Kondo MC Barankevich RA. Vacant lot remediation and firearm violence–a meta-analysis and benefit-to-cost evaluation. Landsc Urban Plan. (2022) 218:104281. doi: 10.1016/j.landurbplan.2021.104281

[13] Frisby JC Kim TWB Schultz EM Adeyemo A Lo KW Hazelton JP. Novel policing techniques decrease gun-violence and the cost to the healthcare system. Prev Med Rep. (2019) 16:100995. doi: 10.1016/j.pmedr.2019.100995, PMID: 31763160 PMC6861592

[14] van Dyke ME Chen MS Sheppard M Sharpe JD Radhakrishnan L Dahlberg LL. County-level social vulnerability and emergency department visits for firearm injuries −10 U.S. jurisdictions, January 1, 2018-December 31, 2021. MMWR Morb Mortal Wkly Rep. (2022) 71:873–7. doi: 10.15585/mmwr.mm7127a1, PMID: 35797204 PMC9290382

[15] Polcari AM Hoefer LE Callier K Zakrison TL Rogers SO Henry MCW. Social vulnerability index is strongly associated with urban pediatric firearm violence: an analysis of five major US cities. J Trauma Acute Care Surg. (2023) 95:411–8. doi: 10.1097/TA.0000000000003896, PMID: 36850025

[16] Spitzer S Castillo-Angeles M Thomas A Hey M D’Souza K Jarman MP. Social vulnerability index and firearms: how neighborhood health disparities affect trauma outcomes. Surg Prac Sci. (2022) 11:100130. doi: 10.1016/j.sipas.2022.100130 PMC11749173 39845167

[17] Monuteaux MC Mannix R Fleegler EW Lee LK. Predictors and outcomes of pediatric firearm injuries treated in the emergency department: differences by mechanism of intent. Acad Emerg Med. (2016) 23:790–5. doi: 10.1111/acem.12986, PMID: 27084566

[18] Controller OOT. Mapping Philadelphia’s gun violence crisis. Available at: https://controller.phila.gov/philadelphia-audits/mapping-gun-violence/#/?year=2023&map=13.26%2F39.91784%2F-75.16346 (Accessed February 2023).

[19] Diversitydatakids.org. Child opportunity index 2.0 ZIP code data. Accessed February, 2023. Available at: https://data.diversitydatakids.org/dataset/coi20_zipcodes-child-opportunity-index-2-0-zip-code-data?_external=True (Accessed February 2023).

[20] Swope CB Hernández D Cushing LJ. The relationship of historical redlining with present-day neighborhood environmental and health outcomes: a scoping review and conceptual model. J Urban Health. (2022) 99:959–83. doi: 10.1007/s11524-022-00665-z, PMID: 35915192 PMC9342590

[21] Noelke C Outrich M Baek M Reece J Osypuk TL McArdle N. Connecting past to present: examining different approaches to linking historical redlining to present day health inequities. PloS One. (2022) 17:e0267606. doi: 10.1371/journal.pone.0267606, PMID: 35587478 PMC9119533

[22] Grossman DC Mueller BA Riedy C Dowd MD Villaveces A Prodzinski J. Gun storage practices and risk of youth suicide and unintentional firearm injuries. JAMA. (2005) 293:707–14. doi: 10.1001/jama.293.6.707, PMID: 15701912

[23] Barkin SL Finch SA Ip EH Scheindlin B Craig JA Steffes J. Is office-based counseling about media use, timeouts, and firearm storage effective? Results from a cluster-randomized, controlled trial. Pediatrics. (2008) 122:e15–25. doi: 10.1542/peds.2007-2611, PMID: 18595960 PMC4486046

[24] Nofi CP Roberts BK Cornell E Tijerina M Tussing O Henry MC. Hospital-based violence intervention programs to reduce firearm injuries in children: a scoping review. J Pediatr Surg. (2023) 58:2212–21. doi: 10.1016/j.jpedsurg.2023.04.020, PMID: 37217364

[25] Byrne JP Kaufman E Scantling D Tam V Martin N Raza S. Association between geospatial access to care and firearm injury mortality in Philadelphia. JAMA Surg. (2022) 157:942–9. doi: 10.1001/jamasurg.2022.3677, PMID: 36001304 PMC9403855

[26] Cohen JS Donnelly K Patel SJ Badolato GM Boyle MD McCarter R. Firearms injuries involving young children in the United States during the COVID-19 pandemic. Pediatrics. (2021) 148:e2020042697. doi: 10.1542/peds.2020-042697, PMID: 33850026

[27] Collings AT Farazi M van Arendonk K Fallat ME Minneci PC Sato TT. The COVID-19 pandemic and associated rise in pediatric firearm injuries: a multi-institutional study. J Pediatr Surg. (2022) 57:1370–6. doi: 10.1016/j.jpedsurg.2022.03.034, PMID: 35501165 PMC9001175

